# Supplementary material for: A Benchmark Dataset for Evaluating Practical Performance of Model Quality Assessment of Homology Models
Source: Bioengineering (Basel). 2022 Mar 15;9(3):118. doi: 10.3390/bioengineering9030118 (PMC8945737; doi:10.3390/bioengineering9030118)
Supplement: Supplementary file 1 [file bioengineering-09-00118-s001.zip › bioengineering-1611157-supplementary.pdf]

Supplementary Materials: A benchmark dataset for evaluating practical performance of model quality assessment of homology models

Yuma Takei [1,2], and Takashi Ishida [1,*]

1. Supplementary result

1.1. The details of the constructed datasets

1.1.1. The list of 100 targets of the single-domain dataset

Table S1. List of 100 targets in the single-domain dataset

Target	Length	Class	Experimental method	Num models	max GDT_TS	med GDT_TS	min GDT_TS
2MR9_A	40	All alpha	SOLUTION NMR	90	0.906	0.812	0.700
4G12_A	44	All alpha	X-RAY DIFFRACTION	150	0.938	0.875	0.773
1HTA_A	68	All alpha	X-RAY DIFFRACTION	150	0.956	0.737	0.570
2B5A_A	77	All alpha	X-RAY DIFFRACTION	150	0.912	0.792	0.597
3DMI_A	87	All alpha	X-RAY DIFFRACTION	150	0.983	0.862	0.402
5V93_o	87	All alpha	ELECTRON MICROSCOPY	150	0.954	0.828	0.422
6K9F_B	87	All alpha	ELECTRON MICROSCOPY	150	0.919	0.825	0.402
1RT8_A	102	All alpha	X-RAY DIFFRACTION	150	0.919	0.739	0.529
2WP2_B	110	All alpha	X-RAY DIFFRACTION	150	0.993	0.849	0.718
1F2E_A	121	All alpha	X-RAY DIFFRACTION	150	0.979	0.746	0.506
1G4I_A	123	All alpha	X-RAY DIFFRACTION	150	0.965	0.880	0.740
3JAI_SS	137	All alpha	ELECTRON MICROSCOPY	150	0.936	0.693	0.411
1VLK_A	142	All alpha	X-RAY DIFFRACTION	95	0.849	0.555	0.405
1EXR_A	146	All alpha	X-RAY DIFFRACTION	150	0.781	0.500	0.402
4HRR_H	150	All alpha	X-RAY DIFFRACTION	150	0.903	0.627	0.522
6O8W_g	154	All alpha	ELECTRON MICROSCOPY	150	0.881	0.753	0.563
5XX9_B	157	All alpha	X-RAY DIFFRACTION	150	0.986	0.770	0.584
2VKE_A	162	All alpha	X-RAY DIFFRACTION	114	0.918	0.616	0.479
3M0G_A	283	All alpha	X-RAY DIFFRACTION	150	0.959	0.793	0.657
3O1D_A	298	All alpha	X-RAY DIFFRACTION	150	0.973	0.644	0.502
4EQF_A	312	All alpha	X-RAY DIFFRACTION	81	0.915	0.765	0.400
1KWF_A	363	All alpha	X-RAY DIFFRACTION	150	0.778	0.565	0.404
4RM4_A	375	All alpha	X-RAY DIFFRACTION	150	0.894	0.730	0.636
3TJ3_A	426	All alpha	X-RAY DIFFRACTION	150	0.927	0.846	0.404
1RE5_A	448	All alpha	X-RAY DIFFRACTION	150	0.926	0.608	0.435
1UTI_A	57	All beta	X-RAY DIFFRACTION	150	0.969	0.908	0.469
1I4K_A	72	All beta	X-RAY DIFFRACTION	150	0.976	0.875	0.781
2F2H_A	80	All beta	X-RAY DIFFRACTION	150	0.978	0.847	0.484
1RHF_A	91	All beta	X-RAY DIFFRACTION	150	0.767	0.631	0.411
2V90_C	93	All beta	X-RAY DIFFRACTION	150	0.908	0.829	0.611
1AZ5_A	99	All beta	X-RAY DIFFRACTION	150	0.960	0.907	0.864
2D9Y_A	104	All beta	SOLUTION NMR	150	0.856	0.700	0.486
2UUB_Q	104	All beta	X-RAY DIFFRACTION	150	0.921	0.651	0.546
1V70_A	105	All beta	X-RAY DIFFRACTION	150	0.741	0.621	0.481
2DS4_A	106	All beta	SOLUTION NMR	150	0.811	0.652	0.554
4ZPL_A	107	All beta	X-RAY DIFFRACTION	150	0.946	0.892	0.734
1BQU_A	115	All beta	X-RAY DIFFRACTION	150	0.947	0.646	0.454
1TW4_A	125	All beta	X-RAY DIFFRACTION	150	0.964	0.804	0.648
1RSY_A	126	All beta	X-RAY DIFFRACTION	150	0.952	0.816	0.615
1QNI_A	131	All beta	X-RAY DIFFRACTION	120	0.991	0.466	0.401
2ZJR_H	134	All beta	X-RAY DIFFRACTION	150	0.925	0.763	0.580
5JP5_A	134	All beta	X-RAY DIFFRACTION	150	0.996	0.918	0.612
4V52_BC	147	All beta	X-RAY DIFFRACTION	150	0.872	0.649	0.449
3GM8_A	158	All beta	X-RAY DIFFRACTION	150	0.926	0.749	0.514
1XWN_A	166	All beta	SOLUTION NMR	150	0.756	0.712	0.664
3JB4_B	166	All beta	ELECTRON MICROSCOPY	150	0.955	0.711	0.408
1OTJ_A	281	All beta	X-RAY DIFFRACTION	150	0.835	0.752	0.422
1HAG_E	295	All beta	X-RAY DIFFRACTION	150	0.780	0.703	0.558

The table continue to the next page.

Table S1. (Continued)

Target	Length	Class	Experimental method	Num models	max GDT_TS	med GDT_TS	min GDT_TS
4WE5_A	321	All beta	X-RAY DIFFRACTION	150	0.972	0.718	0.631
1W8O_A	356	All beta	X-RAY DIFFRACTION	122	0.742	0.643	0.405
1S3J_A	66	alpha + beta	X-RAY DIFFRACTION	150	0.961	0.867	0.590
6KNA_A	76	alpha + beta	SOLUTION NMR	150	0.757	0.706	0.556
2UUB_E	81	alpha + beta	X-RAY DIFFRACTION	150	0.985	0.856	0.725
2I2Y_A	86	alpha + beta	SOLUTION NMR	150	0.817	0.727	0.558
6SPF_T	94	alpha + beta	ELECTRON MICROSCOPY	150	0.936	0.761	0.572
5GJH_A	95	alpha + beta	X-RAY DIFFRACTION	150	0.958	0.747	0.508
1WLU_A	116	alpha + beta	X-RAY DIFFRACTION	150	0.927	0.794	0.489
4IOP_B	121	alpha + beta	X-RAY DIFFRACTION	150	0.884	0.800	0.622
1OH0_A	125	alpha + beta	X-RAY DIFFRACTION	150	0.932	0.695	0.422
5DE0_C	135	alpha + beta	X-RAY DIFFRACTION	150	0.954	0.769	0.404
2C21_A	139	alpha + beta	X-RAY DIFFRACTION	150	0.838	0.634	0.439
1AYZ_A	153	alpha + beta	X-RAY DIFFRACTION	150	0.931	0.845	0.657
1IFV_A	155	alpha + beta	X-RAY DIFFRACTION	150	0.889	0.760	0.405
1HV5_A	161	alpha + beta	X-RAY DIFFRACTION	77	0.862	0.758	0.401
1YR0_A	163	alpha + beta	X-RAY DIFFRACTION	150	0.986	0.730	0.517
1DE4_A	178	alpha + beta	X-RAY DIFFRACTION	150	0.806	0.750	0.664
2BWJ_A	192	alpha + beta	X-RAY DIFFRACTION	150	0.875	0.644	0.591
1K28_A	216	alpha + beta	X-RAY DIFFRACTION	150	0.770	0.627	0.512
5Y4E_A	219	alpha + beta	X-RAY DIFFRACTION	150	0.874	0.607	0.486
1IRU_I	220	alpha + beta	X-RAY DIFFRACTION	150	0.925	0.782	0.624
6CD6_A	287	alpha + beta	X-RAY DIFFRACTION	150	0.963	0.745	0.515
5JT8_A	310	alpha + beta	X-RAY DIFFRACTION	150	0.744	0.653	0.532
2V2F_F	384	alpha + beta	X-RAY DIFFRACTION	150	0.972	0.520	0.403
2DKF_A	431	alpha + beta	X-RAY DIFFRACTION	150	0.725	0.607	0.429
6KWQ_A	462	alpha + beta	X-RAY DIFFRACTION	150	0.976	0.894	0.458
1ST9_A	113	alpha / beta	X-RAY DIFFRACTION	150	0.982	0.754	0.440
3CRN_A	121	alpha / beta	X-RAY DIFFRACTION	150	0.916	0.832	0.461
4NYN_A	133	alpha / beta	X-RAY DIFFRACTION	150	0.906	0.559	0.419
2DYK_A	161	alpha / beta	X-RAY DIFFRACTION	150	0.823	0.660	0.485
1UB7_A	172	alpha / beta	X-RAY DIFFRACTION	150	0.909	0.850	0.646
1OHE_A	182	alpha / beta	X-RAY DIFFRACTION	150	0.844	0.561	0.403
2CNW_D	191	alpha / beta	X-RAY DIFFRACTION	150	0.980	0.842	0.679
2P6R_A	202	alpha / beta	X-RAY DIFFRACTION	150	0.771	0.585	0.477
3GFO_A	222	alpha / beta	X-RAY DIFFRACTION	150	0.812	0.718	0.570
2HSZ_A	224	alpha / beta	X-RAY DIFFRACTION	150	0.771	0.603	0.406
4EFH_A	227	alpha / beta	X-RAY DIFFRACTION	150	0.992	0.906	0.401
4NBU_B	243	alpha / beta	X-RAY DIFFRACTION	150	0.963	0.882	0.717
4KE6_E	246	alpha / beta	X-RAY DIFFRACTION	150	0.997	0.587	0.460
1KA9_F	251	alpha / beta	X-RAY DIFFRACTION	145	0.957	0.781	0.520
3HRX_A	254	alpha / beta	X-RAY DIFFRACTION	150	0.891	0.768	0.664
4PNE_B	270	alpha / beta	X-RAY DIFFRACTION	150	0.752	0.529	0.411
4M1Q_B	312	alpha / beta	X-RAY DIFFRACTION	150	0.919	0.829	0.741
5CZJ_B	319	alpha / beta	X-RAY DIFFRACTION	150	0.998	0.559	0.413
2CYB_B	320	alpha / beta	X-RAY DIFFRACTION	150	0.834	0.706	0.419
2YV3_A	328	alpha / beta	X-RAY DIFFRACTION	150	0.895	0.698	0.401
1I0D_A	331	alpha / beta	X-RAY DIFFRACTION	131	0.999	0.751	0.418
1V2F_A	368	alpha / beta	X-RAY DIFFRACTION	150	0.825	0.743	0.601
2GH9_A	378	alpha / beta	X-RAY DIFFRACTION	150	0.794	0.665	0.427
1EA9_C	382	alpha / beta	X-RAY DIFFRACTION	150	0.947	0.782	0.590
3RHH_C	476	alpha / beta	X-RAY DIFFRACTION	150	0.952	0.794	0.632

The first column represents the target ID. The target ID is composed of the PDB ID and the chain name, but does not necessarily refer to the entire chain because the target sequence was selected based on the SCOP domain classification. The second column indicates the sequence length. The third column represents the SCOP structural class based on the secondary structure. The forth column represents the experimental method for structure determination. The fifth column represents the number of predicted structure models for the target. The sixth, seventh, and eighth columns represent the maximum, median, and minimum GDT_TS values of the structure models, respectively.

1.1.2. The list of 100 targets of the multi-domain dataset

The list of 100 targets of the multi-domain dataset selected from PISCES is shown in the table S2.

Table S2. List of 100 targets in the multi-domain dataset

Target	Length	Num domains	Experimental method	Num models	max GDT_TS	med GDT_TS	min GDT_TS
1L3K_A	196	2	X-RAY DIFFRACTION	150	1.000	0.441	0.400
3P2T_A	196	2	X-RAY DIFFRACTION	150	0.741	0.613	0.450
5Y9Z_A	199	2	X-RAY DIFFRACTION	150	0.992	0.725	0.641
4YI8_A	201	2	X-RAY DIFFRACTION	150	0.952	0.635	0.415
3R2Q_A	202	2	X-RAY DIFFRACTION	150	0.898	0.652	0.444
2IBD_A	204	2	X-RAY DIFFRACTION	150	0.874	0.514	0.403
1VI0_A	206	2	X-RAY DIFFRACTION	150	0.995	0.602	0.402
4D5C_A	206	2	X-RAY DIFFRACTION	150	0.963	0.808	0.402
3LYP_B	215	2	X-RAY DIFFRACTION	150	0.985	0.745	0.478
4BVX_A	215	2	X-RAY DIFFRACTION	145	0.959	0.472	0.409
4I8H_A	223	2	X-RAY DIFFRACTION	150	0.983	0.904	0.719
4XH2_G	228	2	X-RAY DIFFRACTION	150	0.934	0.723	0.589
6HOA_A	228	2	X-RAY DIFFRACTION	138	1.000	0.510	0.406
4IW9_A	231	2	X-RAY DIFFRACTION	150	0.798	0.606	0.431
5K7F_A	231	2	X-RAY DIFFRACTION	150	0.725	0.603	0.404
4KQP_A	232	2	X-RAY DIFFRACTION	150	0.950	0.699	0.458
4PQH_A	236	2	X-RAY DIFFRACTION	150	0.924	0.596	0.452
3K6Y_A	237	2	X-RAY DIFFRACTION	150	0.830	0.551	0.404
4EQ9_A	246	2	X-RAY DIFFRACTION	150	0.934	0.648	0.429
5WJP_A	249	2	X-RAY DIFFRACTION	150	0.707	0.568	0.501
4MF5_A	256	2	X-RAY DIFFRACTION	150	0.957	0.745	0.546
4YKI_A	256	2	X-RAY DIFFRACTION	150	0.913	0.797	0.449
3PFG_A	263	2	X-RAY DIFFRACTION	150	0.924	0.483	0.405
1K5N_A	276	2	X-RAY DIFFRACTION	150	0.998	0.929	0.798
4U19_A	276	2	X-RAY DIFFRACTION	150	0.946	0.627	0.533
4CFS_A	287	2	X-RAY DIFFRACTION	140	1.000	0.504	0.402
1X8B_A	289	2	X-RAY DIFFRACTION	150	0.923	0.691	0.516
4WPG_A	289	2	X-RAY DIFFRACTION	150	0.840	0.628	0.417
3WS7_A	306	2	X-RAY DIFFRACTION	150	0.957	0.676	0.471
6G7N_A	318	2	X-RAY DIFFRACTION	150	0.947	0.604	0.400
1FHU_A	320	2	X-RAY DIFFRACTION	134	0.981	0.498	0.404
4EUO_A	320	2	X-RAY DIFFRACTION	150	0.779	0.514	0.400
2IZR_A	330	2	X-RAY DIFFRACTION	150	0.934	0.834	0.419
4EQB_A	330	2	X-RAY DIFFRACTION	150	0.843	0.504	0.400
5GXE_A	331	2	X-RAY DIFFRACTION	150	0.919	0.580	0.415
4NTC_A	335	2	X-RAY DIFFRACTION	150	0.842	0.551	0.402
4YUC_A	335	2	X-RAY DIFFRACTION	150	0.939	0.666	0.517
2XXG_A	336	2	X-RAY DIFFRACTION	92	0.975	0.671	0.401
1R6D_A	337	2	X-RAY DIFFRACTION	150	0.923	0.745	0.633
3IJL_A	338	2	X-RAY DIFFRACTION	150	0.769	0.590	0.519
4LJ6_A	339	2	X-RAY DIFFRACTION	150	0.837	0.661	0.411
5DP2_A	342	2	X-RAY DIFFRACTION	150	0.902	0.640	0.504
4TMX_A	345	2	X-RAY DIFFRACTION	121	0.784	0.697	0.401
1SVS_A	353	2	X-RAY DIFFRACTION	150	0.998	0.881	0.668
1RKX_A	357	2	X-RAY DIFFRACTION	150	0.914	0.581	0.455
3PJ0_A	359	2	X-RAY DIFFRACTION	140	0.949	0.516	0.414
1GU7_A	364	2	X-RAY DIFFRACTION	97	0.717	0.434	0.400
1LC5_A	364	2	X-RAY DIFFRACTION	150	0.786	0.617	0.447
4H27_A	364	2	X-RAY DIFFRACTION	150	0.928	0.634	0.481
1YQD_A	366	2	X-RAY DIFFRACTION	150	0.984	0.719	0.563
2CDC_B	366	2	X-RAY DIFFRACTION	150	0.818	0.508	0.400
3NYT_A	367	2	X-RAY DIFFRACTION	150	0.920	0.796	0.404
2JHF_B	374	2	X-RAY DIFFRACTION	150	0.995	0.866	0.514
4C08_A	382	2	X-RAY DIFFRACTION	150	0.975	0.753	0.400
4HVK_A	382	2	X-RAY DIFFRACTION	150	0.903	0.697	0.449
3KGW_A	393	2	X-RAY DIFFRACTION	150	0.949	0.717	0.451
4JN7_A	395	2	X-RAY DIFFRACTION	150	0.749	0.668	0.591
2HEU_A	401	2	X-RAY DIFFRACTION	116	0.965	0.416	0.400
2DKJ_A	407	2	X-RAY DIFFRACTION	150	0.950	0.902	0.665

The table continue to the next page.

Table S2. (Continued)

Target	Length	Num domains	Experimental method	Num models	max GDT_TS	med GDT_TS	min GDT_TS
5V8S_A	410	2	X-RAY DIFFRACTION	109	0.713	0.645	0.436
4RY1_A	411	2	X-RAY DIFFRACTION	150	0.813	0.464	0.402
3G7Q_A	417	2	X-RAY DIFFRACTION	150	0.863	0.523	0.414
5DVI_A	419	2	X-RAY DIFFRACTION	102	0.909	0.448	0.408
4WLH_A	422	2	X-RAY DIFFRACTION	150	0.921	0.672	0.409
3SQZ_A	425	2	X-RAY DIFFRACTION	150	0.903	0.737	0.401
2W8T_A	427	2	X-RAY DIFFRACTION	150	0.974	0.698	0.401
3PPL_A	427	2	X-RAY DIFFRACTION	150	0.954	0.456	0.401
4R6H_A	428	2	X-RAY DIFFRACTION	77	0.720	0.426	0.400
6D0A_A	431	2	X-RAY DIFFRACTION	150	0.740	0.571	0.455
3PIU_A	435	2	X-RAY DIFFRACTION	150	0.934	0.550	0.431
4C3S_A	445	2	X-RAY DIFFRACTION	150	0.925	0.543	0.429
4JBE_A	445	2	X-RAY DIFFRACTION	75	0.774	0.439	0.400
3QGU_B	449	2	X-RAY DIFFRACTION	150	0.939	0.588	0.481
5IPY_A	453	2	X-RAY DIFFRACTION	80	0.978	0.608	0.514
4G68_A	456	2	X-RAY DIFFRACTION	150	0.729	0.537	0.405
4G8T_A	464	2	X-RAY DIFFRACTION	150	0.999	0.863	0.400
1HM9_B	468	2	X-RAY DIFFRACTION	150	0.913	0.743	0.638
5JRY_A	485	2	X-RAY DIFFRACTION	150	0.992	0.785	0.586
3A09_A	490	2	X-RAY DIFFRACTION	61	0.967	0.682	0.401
5U3A_A	496	2	X-RAY DIFFRACTION	150	0.999	0.770	0.401
3V4C_A	528	2	X-RAY DIFFRACTION	150	0.874	0.530	0.401
5ICQ_A	610	2	X-RAY DIFFRACTION	55	0.825	0.430	0.401
4A5S_A	740	2	X-RAY DIFFRACTION	150	0.975	0.635	0.409
1ZAR_A	282	3	X-RAY DIFFRACTION	100	0.707	0.589	0.417
3VRD_B	401	3	X-RAY DIFFRACTION	150	0.933	0.474	0.401
4RD4_A	415	3	X-RAY DIFFRACTION	150	0.901	0.653	0.406
4EQS_A	437	3	X-RAY DIFFRACTION	150	0.828	0.707	0.402
4DCU_A	456	3	X-RAY DIFFRACTION	70	0.984	0.470	0.419
3DK9_A	478	3	X-RAY DIFFRACTION	150	0.923	0.837	0.711
6RI6_A	498	3	X-RAY DIFFRACTION	150	0.991	0.957	0.432
6FME_B	506	3	X-RAY DIFFRACTION	146	0.720	0.506	0.420
2WSD_A	513	3	X-RAY DIFFRACTION	150	0.749	0.542	0.402
5FS8_A	515	3	X-RAY DIFFRACTION	150	0.973	0.499	0.401
3VQT_A	548	3	X-RAY DIFFRACTION	150	0.900	0.430	0.402
3AJ7_A	589	3	X-RAY DIFFRACTION	150	0.817	0.658	0.411
2BHU_A	602	3	X-RAY DIFFRACTION	125	0.767	0.694	0.400
3AML_A	755	3	X-RAY DIFFRACTION	150	0.963	0.553	0.405
3KLK_A	1039	3	X-RAY DIFFRACTION	150	0.803	0.689	0.613
3NA5_A	570	4	X-RAY DIFFRACTION	150	0.994	0.460	0.400
3UCQ_A	655	4	X-RAY DIFFRACTION	67	0.819	0.416	0.401

The first column represents the ID of the target. The target ID is composed of the PDB ID and the chain name. The second column indicates the sequence length. The third column represents the number of the domains. The fourth column represents the experimental method of structure determination. The fifth column represents the number of predicted structure models in the target. The sixth, seventh, and eighth columns represent the maximum, median, and minimum GDT_TS values of the structure models, respectively.

1.1.3. The distribution of the sequence length

The distribution of the sequence lengths of the targets included in each dataset is shown in Figure S1. Multi-domain targets are composed of multiple domains, therefore the overall sequence length is longer than that of single-domain targets. The minimum sequence length of the single-domain dataset is 40 and the maximum is 476. The minimum sequence length of the multi-domain dataset is 196, and the maximum is 1039.

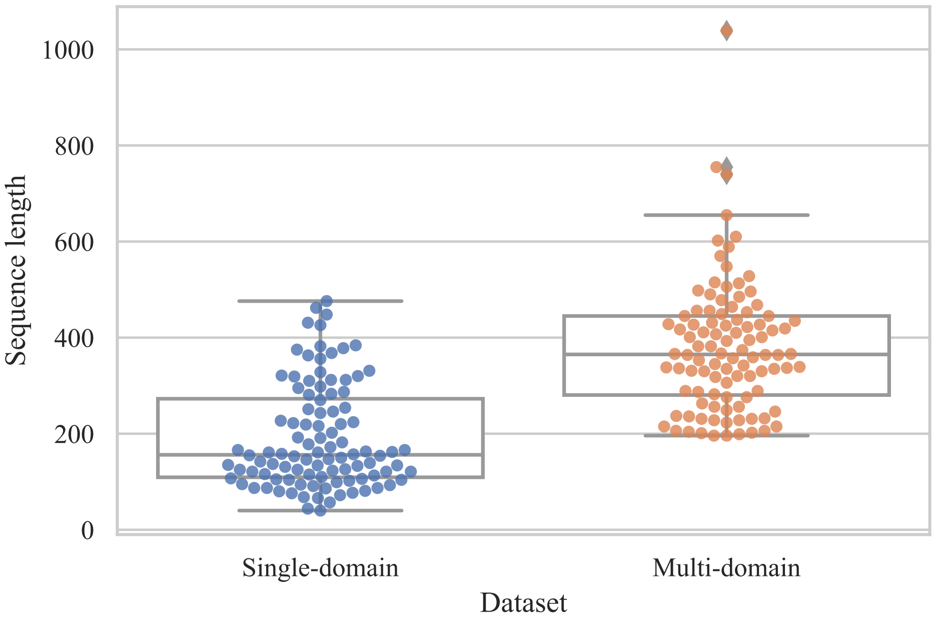

Figure S1. The box plot and swarm plot of the sequence length for each dataset. A single point represents a single target.

1.1.4. The distribution of the GDT_TS of the models for each target

The distribution of GDT_TS of the model for each target in the single-domain dataset is shown in Figure S2, and that of the multi-domain dataset in Figure S3. Most of the targets in the single-domain dataset had an unbiased distribution of GDT_TS. However, for a few targets, such as ’1DA9_C’ and ’1XWN_A’, the range of the distribution of the GDT_TS is narrow. In the multi-domain dataset, for about 20% of the total targets, the models are polarized into a few models with higher GDT_TS and other lower models.

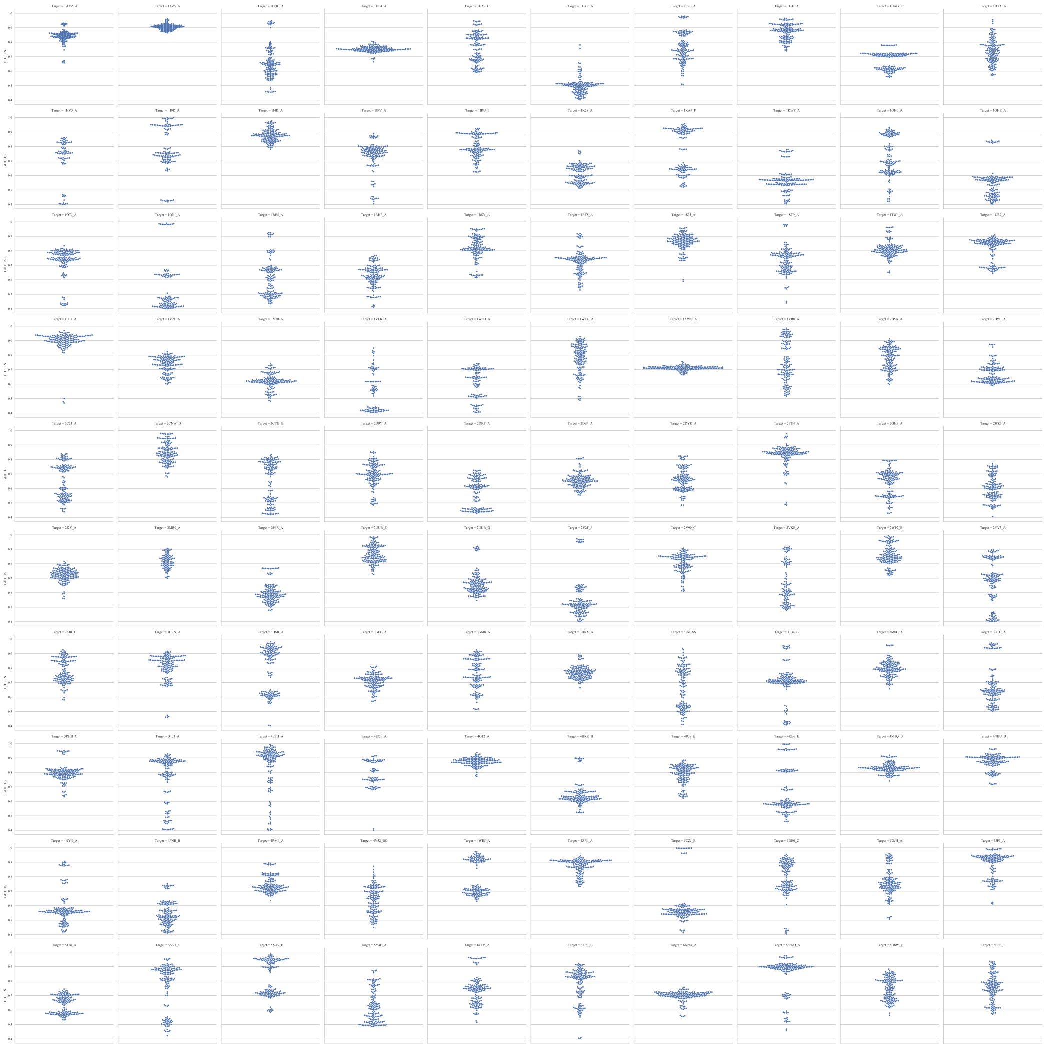

Figure S2. The swarm plot of the GDT_TS for each target of single-domain dataset. A single point represents a single model. The range of GDT_TS in the plot is 0.4 to 1.

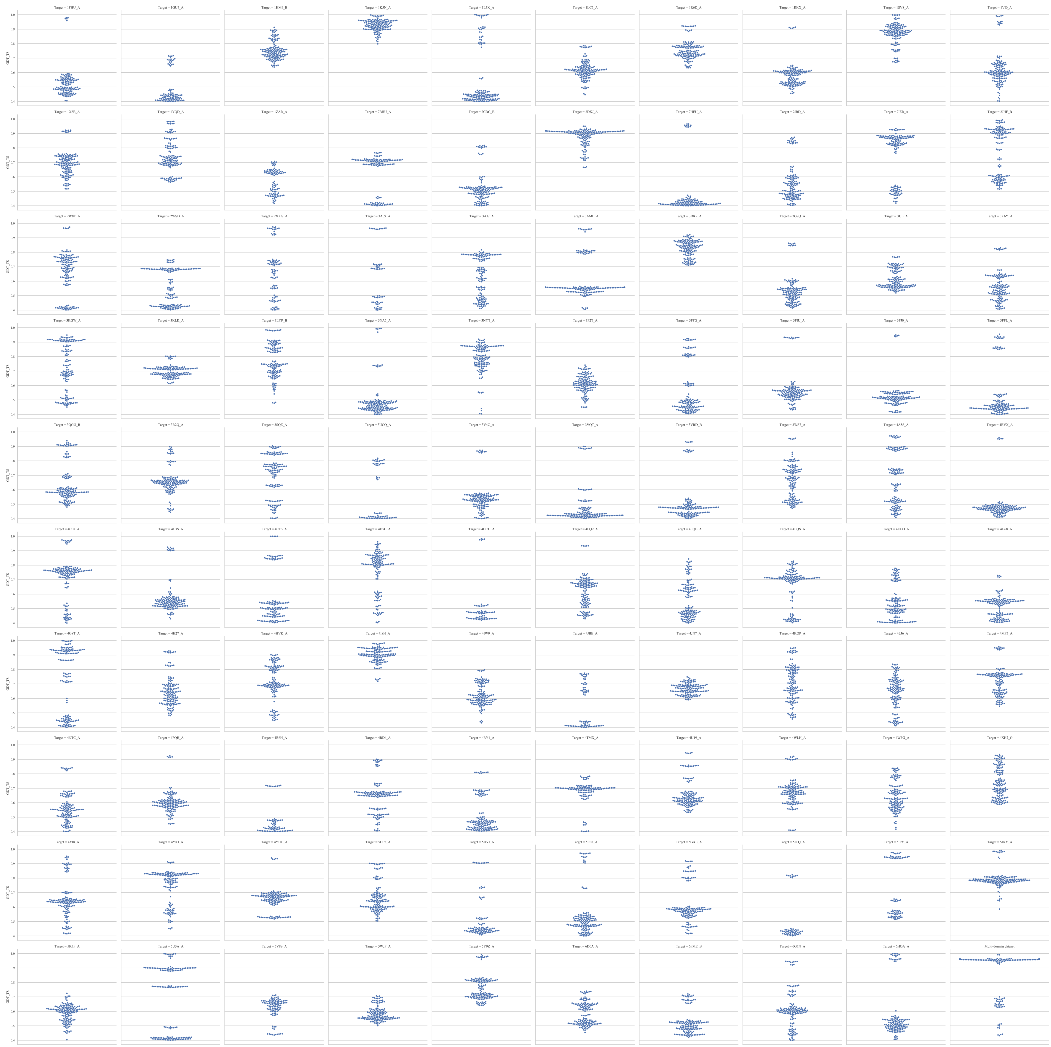

Figure S3. The swarm plot of the GDT_TS for each target of Multi-domain dataset.

1.1.5. Comparison to CASP dataset

We compare the constructed datasets with CASP11-13 [1–3], which are often used as a test dataset in MQA research. The CASP dataset for MQA is divided into stage1 and stage2, and stage2 is used in this comparison.

The results of the comparison for each dataset regarding the number of targets with the maximum GDT_TS value above the threshold are shown in Table S3. From this table, we can see that the number of targets with GDT_TS greater than 0.7 do not differ significantly between the three datasets, but as we increase the threshold of GDT_TS, the number of targets in the CASP dataset is less than that in the constructed datasets. In particular, for the number of targets with GDT_TS greater than 0.9, there is a considerable difference between the constructed datasets and the CASP dataset. Therefore, we were able to construct the datasets with more targets that contain high accurate models compared to the CASP dataset.

Table S3. Number of targets for which the maximum GDT_TS value exceeds the threshold in each dataset

Dataset	All	Threshold of the maximum GDT_TS		
		0.7	0.8	0.9
Single-domain	100	100	85	63
Multi-domain	100	100	81	64
CASP11-13	239	87	54	19

The first column indicates the dataset name. The second column represents the number of targets in the entire dataset. Columns 3 to 5 show the number of targets for which the maximum GDT_TS exceeds the threshold.

1.1.6. Differences in the quality of the models with the same template

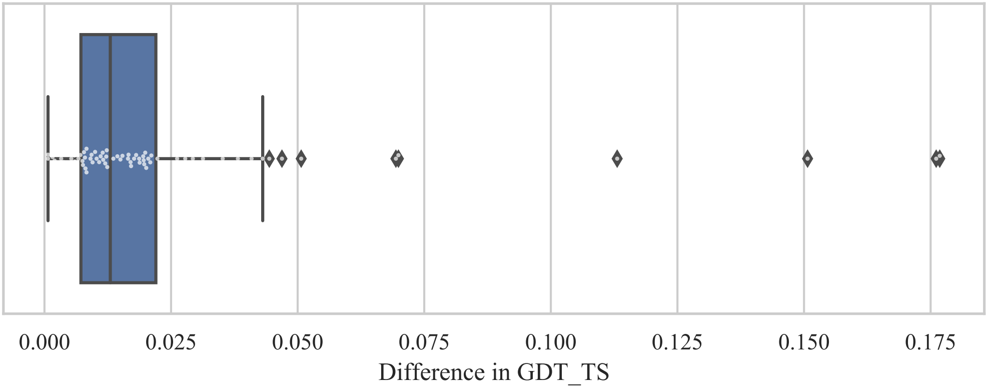

Figure S4. The box plot and the swarm plot of the difference in GDT_TS between the best and the worst models from the same template and alignment for the single-domain dataset. Only the template of the model with the highest GDT_TS in each target is shown. A white point represents a template.

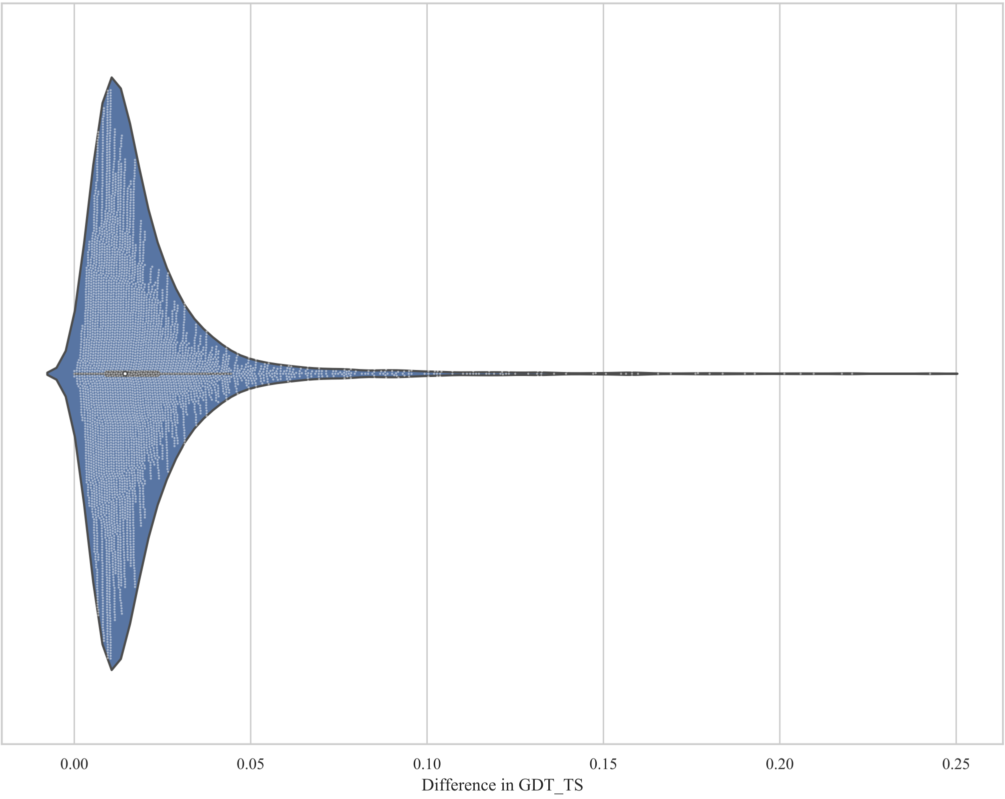

Figure S5. The violin plot and the swarm plot of the difference in GDT_TS between the best and the worst models from the same template and alignment for the single-domain dataset. A white point represents a template.

1.2. MQA Performance evaluation for the constructed dataset

Table S4. MQA performance for the single-domain dataset

Method	Loss	MAE	Pearson	Spearman
identity(%)	4.096<br>(-)	(0.371)<br>(-)	0.636<br>(-)	0.507<br>(-)
positive(%)	4.902<br>(2.87×10^{-01})	(0.215)<br>($\mathbf{7.79 \times 10^{-18}}$)	0.661<br>($\mathbf{1.65 \times 10^{-05}}$)	0.540<br>($\mathbf{8.25 \times 10^{-05}}$)
coverage(%)	10.068<br>($\mathbf{3.46 \times 10^{-10}}$)	(0.211)<br>($\mathbf{3.12 \times 10^{-13}}$)	0.438<br>($\mathbf{7.00 \times 10^{-08}}$)	0.359<br>($\mathbf{6.88 \times 10^{-06}}$)
DOPE	4.013<br>(7.39×10^{-01})	-<br>(-)	0.745<br>($\mathbf{4.75 \times 10^{-05}}$)	0.675<br>($\mathbf{1.11 \times 10^{-09}}$)
SOAP	3.818<br>(3.36×10^{-01})	-<br>(-)	0.642<br>(8.47×10^{-01})	0.603<br>($\mathbf{3.86 \times 10^{-05}}$)
ProQ3D	4.562<br>(7.18×10^{-01})	0.129<br>($\mathbf{1.46 \times 10^{-17}}$)	0.725<br>($\mathbf{2.10 \times 10^{-04}}$)	0.663<br>($\mathbf{4.03 \times 10^{-09}}$)
SBROD	5.797<br>(2.30×10^{-02})	-<br>(-)	0.676<br>(6.58×10^{-02})	0.613<br>($\mathbf{5.58 \times 10^{-05}}$)
P3CMQA	**3.091**<br>(1.70×10^{-02})	**0.096**<br>($\mathbf{6.50 \times 10^{-18}}$)	**0.838**<br>($\mathbf{4.56 \times 10^{-13}}$)	**0.777**<br>($\mathbf{5.05 \times 10^{-15}}$)
DeepAccNet	3.288<br>(2.97×10^{-02})	0.238<br>($\mathbf{6.66 \times 10^{-16}}$)	0.748<br>($\mathbf{9.77 \times 10^{-07}}$)	0.675<br>($\mathbf{3.30 \times 10^{-11}}$)
DeepAccNet-Bert	3.372<br>(2.78×10^{-02})	0.173<br>($\mathbf{2.79 \times 10^{-17}}$)	0.821<br>($\mathbf{3.01 \times 10^{-11}}$)	0.754<br>($\mathbf{5.49 \times 10^{-14}}$)

The first column represents the method name. The second column shows the average GDT_TS loss of the selected models for each target. The values are multiplied by 100 for clarity. The third column shows the average mean absolute error (MAE) between the GDT_TS and estimated scores per target. The fourth and fifth columns show the average Pearson and Spearman correlation coefficients for each target, respectively. The values in parentheses in the second line of each method are the p-values (calculated by the Wilcoxon signed-rank test against identity). The MAE values for identity, positive, and coverage are given in parentheses because they are not scores that directly predict the quality of the model structures. The best values are in bold.

Table S5. MQA performance for the multi-domain dataset

Method	Loss	MAE	Pearson	Spearman
identity(%)	4.885<br>(-)	(0.318)<br>(-)	0.787<br>(-)	0.551<br>(-)
positive(%)	4.410<br>(4.75×10^{-02})	(0.171)<br>(**4.27×10^{-18}**)	0.805<br>(**1.51×10^{-03}**)	0.577<br>(**3.14×10^{-03}**)
coverage(%)	16.252<br>(**6.64×10^{-10}**)	(0.285)<br>(1.72×10^{-02})	0.424<br>(**2.40×10^{-16}**)	0.387<br>(**1.52×10^{-05}**)
DOPE	2.468<br>(**3.07×10^{-03}**)	-<br>(-)	0.809<br>(2.61×10^{-01})	0.712<br>(**4.08×10^{-08}**)
SOAP	2.921<br>(**1.03×10^{-03}**)	-<br>(-)	0.741<br>(**1.17×10^{-03}**)	0.620<br>(**9.15×10^{-03}**)
ProQ3D	3.587<br>(1.65×10^{-02})	0.095<br>(**8.27×10^{-18}**)	0.817<br>(7.55×10^{-02})	0.723<br>(**7.55×10^{-08}**)
SBROD	3.684<br>(6.06×10^{-02})	-<br>(-)	0.785<br>(2.88×10^{-01})	0.676<br>(**8.14×10^{-05}**)
P3CMQA	**1.884**<br>(**4.29×10^{-05}**)	**0.075**<br>(**7.56×10^{-18}**)	**0.884**<br>(**4.90×10^{-11}**)	**0.802**<br>(**2.34×10^{-15}**)
DeepAccNet	2.873<br>(2.52×10^{-02})	0.194<br>(**2.71×10^{-17}**)	0.858<br>(**6.35×10^{-09}**)	0.734<br>(**1.31×10^{-10}**)
DeepAccNet-Bert	2.760<br>(**7.28×10^{-04}**)	0.142<br>(**1.41×10^{-17}**)	0.882<br>(**4.66×10^{-10}**)	0.788<br>(**6.60×10^{-14}**)

The legends are the same as those in Table S4.

1.3. MQA Performance evaluation when RMSD is used as a label

The results of the MQA performance when RMSD is used as a label are shown in Table S6 and S7. RMSD was calculated using PyMOL[4].

The performance of the deep learning-based method was the best, as it was when GDT_TS was used as a label. However, on the single domain dataset, identity and DOPE were not significantly different from the deep learning-based method for loss.

Table S6. MQA performance for the single-domain dataset with RMSD as a label

Method	RMSD Loss	RMSD Pearson	RMSD Spearman
identity(%)	-0.290	-0.506	-0.484
positive(%)	-0.515	-0.529	-0.491
coverage(%)	-0.947	-0.334	-0.277
DOPE	-0.290	-0.629	-0.586
SOAP	-0.353	-0.555	-0.559
ProQ3D	-0.417	-0.603	-0.572
SBROD	-0.497	-0.559	-0.535
P3CMQA	-0.310	**-0.692**	-0.645
DeepAccNet	**-0.227**	-0.621	-0.613
DeepAccNet-Bert	-0.368	-0.686	**-0.664**

The first column represents the method name. The second column shows the average RMSD loss of the selected models for each target. The smaller the RMSD is, the less difference there is between the predicted structure and the ground truth structure, thus loss takes a negative value, and the closer to zero it is, the better. The third and fourth columns show the average Pearson and Spearman correlation coefficients for each target, respectively. Similar to loss, Pearson and Spearman also take negative values, and larger absolute values indicate stronger correlation. The best values are in bold.

Table S7. MQA performance for the multi-domain dataset with RMSD as a label

Method	RMSD Loss	RMSD Pearson	RMSD Spearman
identity(%)	-0.420	-0.673	-0.534
positive(%)	-0.392	-0.690	-0.547
coverage(%)	-1.326	-0.372	-0.312
DOPE	-0.188	-0.702	-0.623
SOAP	-0.228	-0.632	-0.560
ProQ3D	-0.314	-0.711	-0.634
SBROD	-0.354	-0.689	-0.600
P3CMQA	**-0.154**	**-0.769**	**-0.705**
DeepAccNet	-0.223	-0.730	-0.665
DeepAccNet-Bert	-0.218	-0.759	-0.694

The legends are the same as those in Table S6.

1.4. Situation-specific MQA performance

Table S8. MQA performance for each category based on the distribution of identity for the single-domain dataset

Category	Num targets	Method	Loss	Pearson	Spearman
Single top	9	identity(%)	**1.900**	0.709	0.511
		positive(%)	**1.900**	0.734	0.554
		coverage(%)	9.505	0.618	0.432
		DOPE	3.213	0.886	0.741
		SOAP	2.862	0.782	0.697
		ProQ3D	4.348	0.877	0.744
		SBROD	3.390	0.813	0.698
		P3CMQA	3.833	**0.926**	**0.821**
		DeepAccNet	3.573	0.855	0.757
		DeepAccNet-Bert	2.539	0.914	0.808
Multi top	41	identity(%)	3.177	0.661	0.481
		positive(%)	3.659	0.693	0.533
		coverage(%)	9.828	0.445	0.360
		DOPE	3.499	0.735	0.665
		SOAP	3.276	0.641	0.591
		ProQ3D	4.064	0.699	0.660
		SBROD	6.046	0.658	0.597
		P3CMQA	2.459	**0.822**	**0.769**
		DeepAccNet	**2.070**	0.752	0.671
		DeepAccNet-Bert	3.159	0.817	0.752
No identical top	50	identity(%)	5.244	0.602	0.528
		positive(%)	6.461	0.623	0.542
		coverage(%)	10.366	0.400	0.345
		DOPE	4.578	0.727	0.672
		SOAP	4.435	0.618	0.596
		ProQ3D	5.008	0.718	0.651
		SBROD	6.026	0.666	0.611
		P3CMQA	**3.475**	**0.836**	**0.775**
		DeepAccNet	4.237	0.725	0.664
		DeepAccNet-Bert	3.697	0.807	0.745

The first column represents the category name based on the distribution of sequence identity. The second column shows the number of the targets for each category. The third column shows the method name. The fourth, fifth, and sixth columns represent GDT_TS loss, Pearson correlation coefficients, Spearman correlation coefficients. The best values in each category are shown in bold.

Table S9. MQA performance for each category based on the distribution of identity for the multi-domain dataset

Category	Num targets	Method	Loss	Pearson	Spearman
Single top	20	identity(%)	0.751	0.829	0.410
		positive(%)	0.751	0.837	0.452
		coverage(%)	16.930	0.470	0.400
		DOPE	1.703	0.857	0.702
		SOAP	**0.691**	0.779	0.565
		ProQ3D	4.221	0.829	0.694
		SBROD	2.178	0.794	0.675
		P3CMQA	0.890	0.895	**0.798**
		DeepAccNet	1.102	0.901	0.722
		DeepAccNet-Bert	0.972	**0.920**	**0.798**
Multi top	34	identity(%)	3.067	0.854	0.575
		positive(%)	2.900	0.867	0.609
		coverage(%)	18.513	0.417	0.389
		DOPE	2.494	0.838	0.723
		SOAP	2.317	0.779	0.634
		ProQ3D	3.299	0.848	0.743
		SBROD	2.778	0.824	0.691
		P3CMQA	**2.275**	0.906	**0.810**
		DeepAccNet	3.129	0.910	0.750
		DeepAccNet-Bert	2.624	**0.925**	0.803
No identical top	46	identity(%)	8.026	0.720	0.595
		positive(%)	7.118	0.746	0.608
		coverage(%)	14.286	0.410	0.379
		DOPE	2.783	0.768	0.708
		SOAP	4.338	0.696	0.633
		ProQ3D	3.525	0.790	0.722
		SBROD	5.009	0.753	0.666
		P3CMQA	**2.027**	**0.862**	**0.797**
		DeepAccNet	3.453	0.800	0.728
		DeepAccNet-Bert	3.639	0.833	0.773

The legends are the same as those in Table S8.

Table S10. MQA performance for each category based on the maximum identity for the single-domain dataset

Category	Num targets	Method	Loss	Pearson	Spearman
High	24	identity(%)	**4.357**	0.726	0.581
		positive(%)	4.788	0.751	0.607
		coverage(%)	10.483	0.472	0.390
		DOPE	5.675	0.711	0.611
		SOAP	5.667	0.670	0.585
		ProQ3D	6.376	0.659	0.605
		SBROD	7.622	0.627	0.546
		P3CMQA	5.377	**0.814**	**0.731**
		DeepAccNet	5.014	0.732	0.636
		DeepAccNet-Bert	5.760	0.762	0.672
Mid-high	23	identity(%)	3.544	0.652	0.498
		positive(%)	4.014	0.682	0.536
		coverage(%)	8.516	0.489	0.408
		DOPE	3.428	0.782	0.725
		SOAP	3.157	0.685	0.660
		ProQ3D	4.543	0.753	0.703
		SBROD	4.497	0.716	0.680
		P3CMQA	**1.981**	**0.866**	**0.814**
		DeepAccNet	2.336	0.776	0.712
		DeepAccNet-Bert	3.056	0.848	0.781
Mid-low	39	identity(%)	3.969	0.623	0.504
		positive(%)	4.679	0.643	0.541
		coverage(%)	10.736	0.404	0.318
		DOPE	3.345	0.738	0.678
		SOAP	2.876	0.626	0.595
		ProQ3D	4.413	0.731	0.669
		SBROD	6.078	0.678	0.607
		P3CMQA	2.695	0.825	0.772
		DeepAccNet	2.758	0.731	0.659
		DeepAccNet-Bert	**2.598**	**0.830**	**0.773**
Low	14	identity(%)	4.908	0.491	0.402
		positive(%)	7.175	0.525	0.426
		coverage(%)	10.044	0.392	0.341
		DOPE	3.987	0.761	0.696
		SOAP	4.357	0.570	0.561
		ProQ3D	**1.896**	0.771	0.681
		SBROD	4.020	0.688	0.636
		P3CMQA	2.096	**0.870**	**0.808**
		DeepAccNet	3.373	0.776	0.725
		DeepAccNet-Bert	1.954	0.851	0.796

The first column represents the name of the category based on the maximum value of identity per target. The second column shows the number of the targets for each category. The third column shows the method name. The fourth, fifth, and sixth columns represent GDT_TS loss, Pearson correlation coefficients, Spearman correlation coefficients.

Table S11. MQA performance for each category based on the maximum identity for the multi-domain dataset

Category	Num targets	Method	Loss	Pearson	Spearman
High	31	identity(%)	3.290	0.811	0.487
		positive(%)	2.680	0.824	0.513
		coverage(%)	17.769	0.459	0.379
		DOPE	2.860	0.823	0.711
		SOAP	1.935	0.807	0.660
		ProQ3D	5.325	0.807	0.700
		SBROD	4.539	0.772	0.656
		P3CMQA	**2.495**	0.864	**0.768**
		DeepAccNet	2.592	0.867	0.740
		DeepAccNet-Bert	2.941	**0.873**	0.756
Mid-high	22	identity(%)	2.751	0.836	0.649
		positive(%)	2.778	0.843	0.664
		coverage(%)	10.589	0.520	0.495
		DOPE	1.818	0.859	0.756
		SOAP	1.919	0.777	0.630
		ProQ3D	2.300	0.842	0.727
		SBROD	3.266	0.803	0.702
		P3CMQA	**1.353**	**0.908**	**0.818**
		DeepAccNet	2.198	0.874	0.733
		DeepAccNet-Bert	2.452	0.890	0.802
Mid-low	30	identity(%)	6.620	0.808	0.567
		positive(%)	6.031	0.809	0.578
		coverage(%)	18.288	0.392	0.375
		DOPE	2.721	0.809	0.694
		SOAP	3.364	0.742	0.610
		ProQ3D	2.728	0.828	0.740
		SBROD	2.491	0.799	0.680
		P3CMQA	**2.106**	0.883	**0.802**
		DeepAccNet	3.103	0.867	0.717
		DeepAccNet-Bert	2.882	**0.894**	0.793
Low	17	identity(%)	7.493	0.645	0.513
		positive(%)	6.819	0.717	0.580
		coverage(%)	17.220	0.293	0.281
		DOPE	2.152	0.722	0.688
		SOAP	5.235	0.570	0.551
		ProQ3D	3.599	0.787	0.733
		SBROD	4.772	0.764	0.675
		P3CMQA	**1.065**	**0.891**	**0.842**
		DeepAccNet	3.851	0.802	0.755
		DeepAccNet-Bert	2.614	0.866	0.819

The first column represents the name of the category based on the maximum value of identity per target. The second column shows the number of the targets for the category. The third column shows the method name. The fourth, fifth, and sixth columns represent GDT_TS loss, Pearson correlation coefficients, Spearman correlation coefficients.

Table S12. MQA performance for each class of the single-domain dataset

Class	Num targets	Method	Loss	MAE	Pearson	Spearman
All alpha	25	identity(%)	5.518	0.378	0.662	0.568
		positive(%)	5.820	0.231	0.693	0.600
		coverage(%)	9.829	0.206	0.475	0.462
		DOPE	4.795	-	0.702	0.664
		SOAP	3.866	-	0.615	0.577
		ProQ3D	6.134	0.178	0.652	0.607
		SBROD	5.898	-	0.642	0.595
		P3CMQA	4.489	0.106	0.805	0.762
		DeepAccNet	3.159	0.265	0.702	0.634
		DeepAccNet-Bert	3.658	0.190	0.768	0.722
All beta	25	identity(%)	3.289	0.374	0.604	0.449
		positive(%)	4.563	0.217	0.638	0.504
		coverage(%)	8.740	0.192	0.478	0.315
		DOPE	3.780	-	0.754	0.663
		SOAP	4.271	-	0.623	0.607
		ProQ3D	3.094	0.122	0.716	0.652
		SBROD	6.379	-	0.634	0.566
		P3CMQA	2.280	0.095	0.853	0.792
		DeepAccNet	3.458	0.230	0.754	0.681
		DeepAccNet-Bert	4.279	0.170	0.827	0.749
alpha + beta	25	identity(%)	4.098	0.369	0.544	0.428
		positive(%)	5.567	0.213	0.582	0.454
		coverage(%)	11.477	0.209	0.436	0.340
		DOPE	3.326	-	0.753	0.671
		SOAP	3.049	-	0.637	0.591
		ProQ3D	5.402	0.123	0.752	0.673
		SBROD	6.072	-	0.709	0.645
		P3CMQA	3.356	0.100	0.837	0.763
		DeepAccNet	3.554	0.226	0.739	0.674
		DeepAccNet-Bert	2.533	0.170	0.827	0.748
alpha / beta	25	identity(%)	3.479	0.365	0.733	0.583
		positive(%)	3.657	0.199	0.733	0.601
		coverage(%)	10.225	0.237	0.364	0.318
		DOPE	4.152	-	0.769	0.702
		SOAP	4.086	-	0.694	0.637
		ProQ3D	3.616	0.095	0.779	0.720
		SBROD	4.840	-	0.718	0.646
		P3CMQA	2.237	0.084	0.859	0.790
		DeepAccNet	2.983	0.232	0.797	0.710
		DeepAccNet-Bert	3.019	0.162	0.860	0.796

The first column represents the name of the class defined in SCOP. The second column shows the number of the targets.

Table S13. MQA performance for each number of domains of the multi-domain dataset

Num domains	Num targets	Method	Loss	MAE	Pearson	Spearman
2	83	identity(%)	4.910	0.327	0.769	0.542
		positive(%)	4.328	0.177	0.791	0.571
		coverage(%)	16.223	0.281	0.402	0.366
		DOPE	2.338	-	0.803	0.706
		SOAP	2.807	-	0.733	0.613
		ProQ3D	3.017	0.096	0.814	0.723
		SBROD	3.892	-	0.777	0.678
		P3CMQA	1.736	0.075	0.877	0.799
		DeepAccNet	2.829	0.198	0.852	0.730
		DeepAccNet-Bert	2.736	0.138	0.877	0.790
3	15	identity(%)	5.063	0.282	0.874	0.591
		positive(%)	5.123	0.143	0.871	0.604
		coverage(%)	14.947	0.296	0.543	0.485
		DOPE	3.289	-	0.834	0.759
		SOAP	3.627	-	0.760	0.651
		ProQ3D	7.132	0.095	0.816	0.724
		SBROD	2.608	-	0.812	0.671
		P3CMQA	2.955	0.078	0.906	0.815
		DeepAccNet	3.032	0.176	0.874	0.756
		DeepAccNet-Bert	2.798	0.164	0.896	0.781
4	2	identity(%)	2.486	0.241	0.906	0.622
		positive(%)	2.486	0.111	0.907	0.614
		coverage(%)	27.234	0.376	0.449	0.497
		DOPE	1.740	-	0.897	0.599
		SOAP	2.365	-	0.919	0.648
		ProQ3D	0.655	0.090	0.969	0.735
		SBROD	3.145	-	0.936	0.646
		P3CMQA	0.000	0.068	0.977	0.821
		DeepAccNet	3.490	0.176	0.966	0.741
		DeepAccNet-Bert	3.490	0.149	0.964	0.752

The first column represents the number of the domains. The second column shows the number of the targets.

References

1. Moult, J.; Fidelis, K.; Kryshtafovych, A.; Schwede, T.; Tramontano, A. Critical assessment of methods of protein structure prediction: Progress and new directions in round XI. *Proteins: Structure, Function, and Bioinformatics* **2016**, *84*, 4–14. doi:10.1002/prot.25064.
2. Moult, J.; Fidelis, K.; Kryshtafovych, A.; Schwede, T.; Tramontano, A.; Topf, M.; Fidelis, K.; Moult, J.; Fidelis, K.; Kryshtafovych, A.; et al. Critical assessment of methods of protein structure prediction (CASP)—Round XII. *Proteins: Structure, Function, and Bioinformatics* **2018**, *86*, 7–15. doi:10.1002/prot.25415.
3. Kryshtafovych, A.; Schwede, T.; Topf, M.; Fidelis, K.; Moult, J. Critical assessment of methods of protein structure prediction (CASP)—Round XIII. *Proteins: Structure, Function, and Bioinformatics* **2019**, *87*, 1011–1020. doi:10.1002/prot.25823.
4. Schrödinger, LLC. The PyMOL Molecular Graphics System, Version 1.8.
